# Effect of Alumina Nanowires on the Thermal Conductivity and Electrical Performance of Epoxy Composites

**DOI:** 10.3390/polym12092126

**Published:** 2020-09-17

**Authors:** Liangsong Huang, Xitao Lv, Yongzhe Tang, Guanghui Ge, Peng Zhang, Yuxia Li

**Affiliations:** Key Laboratory for Robot Intelligent Technology of Shandong Province, Shandong University of Science and Technology, Qingdao 266590, China; lshuang@sdust.edu.cn (L.H.); xtlv@sdust.edu.cn (X.L.); yztang@sdust.edu.cn (Y.T.); ghge@sdust.edu.cn (G.G.); pengzhang@sdust.edu.cn (P.Z.)

**Keywords:** Al_2_O_3_-NWs, composite, thermal conductivity, volume resistivity, dielectric performance, breakdown strength

## Abstract

Alumina nanowires (Al_2_O_3_-NWs)/epoxy resin composites have been thoroughly studied due to their excellent insulating and dielectric performance. In particular, understanding the effect of nano-alumina with different morphologies on the dielectric performance of composites is of great significance. In this study, Al_2_O_3_-NWs with lengths of approximately 100 nm and diameters of approximately 5 nm were prepared and blended with anepoxy resin to form composites, and the effect of the mass fraction of fillers on the thermal conductivity of the composites was investigated. Specifically, the effect of alumina fillers with ananowire structure on the insulating and dielectric performance and breakdown strength of the epoxy composites were analyzed. The influence principle of the interfacial effect and heat accumulation on the dielectric and insulating properties of the composites were described. The results demonstrated that the thermal conductivity of Al_2_O_3_-NWs/epoxy resin composites was higher than that of the bare epoxy resin. The thermal conductivity of Al_2_O_3_-NWs/epoxy resin composites increased with increasing mass fraction of fillers. When the mass fraction of fillers was 10%, the thermal conductivity of the composite was 134% higher than that of the epoxy resin matrix. The volume resistivity of the composites first increased and then decreased as the mass fraction of fillers increased, while the dielectric constant of the composites increased with increasing mass fraction of fillers and decreasing frequency. The dielectric loss of the composites decreased and then increased as the mass fraction of fillers increased, and it increased with increasing frequency. Additionally, the alternating current breakdown strength of the composites first increased and then decreased withincreasingmass fraction of fillers.

## 1. Introduction

Epoxy resins have excellent mechanical and insulation performance and are widely used in the field of electrical and electronic information. The breakdown strength of epoxy resins is as high as 28 KV/mm, the dielectric constant at power frequency is approximately 3.5, and the mechanical elasticity can reach 53 GPa [1,2]. The integration of electronics and chips has been increasing due to the development of electrical and electronic technology, which generates increased heat accumulation within the electronic devices. This results in higher environmental temperatures in electrical equipment during operation, which not only reduces the stability of the equipment but also reduces its service life. Hence, it is especially important to improve the thermal conductivity of insulating materials [3,4]. Moreover, the voltage level of power equipment has gradually increased, which also requires better electrical performance of insulating materials [5,6,7]. Epoxy resins, which are limited by low thermal conductivity, high thermal expansion, and poor polarization performance, fail to meet the requirements for their application in electrical equipment. Therefore, the addition of inorganic nano-fillers to epoxy resins has been widely studied in recent yearsto enhance their thermal conductivity and electrical performance [8,9,10]. For example, alumina has been considered an interesting material to be used as a filler. The coefficient of thermal conductivity of alumina is approximately 30 w/mK, its resistivity is between 10^12^ and 10^14^ Ω, and it has good thermal conductivity and insulation performance compared with pure epoxy resins [11,12]. The presence of alumina fillers in an epoxy resin matrix enhances the thermal conductivity of composites. Due to the high dielectric constant of the fillers, the dielectric constant of the composite increases, and the breakdown strength of the composite will also be improved to a certain extent, which can provide higher power or storage density [13]. However, nanoparticles have a variety of morphological characteristics, and the different morphologies can have different effects on composite properties [14,15]. At present, there are only a few studies on the effect of nanoparticles with different morphologies on the electrical performance of epoxy resin composites. Consequently, studying the influence of nano-alumina with different morphologies on the electrical performance of composites has a great significance for practical applications.

Kochetovet al. studied the thermal conductivity of epoxy composites with inorganic nanoparticles such as alumina. The results showed that the thermal conductivity of Al_2_O_3_-NWs/epoxy composites went up with increasing mass fraction of alumina. Additionally, two- and three-phase Lewis–Nielsen models were proposed to specifically solve the effect of interfacial characteristics between the polymer matrix and the filler nanoparticles on the thermal conductivity of the composites [16]. Moreira et al. studied the effect of size on the thermal conductivity of nano-composites filled with alumina nanoparticles. Nano-alumina particles with diameters of 30–40, 27–43, 150, and 200 nm were added to the epoxy resin matrix. It was found that the nanoparticles with a larger particle size had a stronger agglomeration and a better thermal conductivity enhancement compared with nano-fillers with small particle sizes. The study also revealed that interface characteristics played an important role in thermal conductivity [17]. Anithambigai et al. studied the effect of size on the thermal conductivity of epoxy composites with alumina. The results showed that the thermal conductivity of the composites with 44 µm and 10 µm alumina particles were improved by 2.06 and 1.19 W/mK, respectively, compared to the pure epoxy resin [18]. Eker et al. studied the effect of inorganic metal oxide fillers (e.g., alumina) on the resistivity of epoxy resins by changing the potential and environmental temperature applied to the composite. It was found that the thermal stability of the composite was enhanced, and the charge accommodation between the fillers and the matrix improved its resistivity. However, the volume resistivity of the composite was slightly lower than that of the pure epoxy resin, and as the temperature increased to 370 K, the resistivity of the composite decreased [19]. Tomaskova et al. investigated the effect of microscale alumina fillers on the thermal conductivity and dielectric performance of epoxy composites. It was found that the volume resistivity of the composites with micro-alumina fillers was improved, while no significant differences were observed for dielectric loss. Overall, micro-fillers haven been shown to significantly improve the thermal conductivity of materials [20]. Paul et al. studied the enhancement of the dielectric performance of composites with different doping ratios of nano-alumina fillers. It was determined that the optimal doping ratio was obtained with particle sizes of 70 and 40 nm. However, the breakdown strength of the composite was lower than that of the pure epoxy resin [21]. Gong et al. investigated the effect of aluminum (Al) with binary particle size distribution on the mechanical and dielectric performance of epoxy composites. It was found that after adding Al in the appropriate mass fraction, the mechanical and dielectric performance of the composites were enhanced by the synergistic effect of the hybrid particles [22]. Moreover, Ramu et al. investigated the effect of nano-alumina on the insulating performance of epoxy composites. The dielectric performance of the composite was enhanced by adding fillers with an appropriate doping ratio, and the composite breakdown strength as a function of filler doping ratio was studied [23]. Nascimento et al. analyzedthe effect of nano-alumina fillers on the breakdown performance of epoxy composites. It was reported that at low doping ratios, charges were trapped by the interaction between the filler nanoparticles and the epoxy particles, creatinga diffused electric double layer, which hindered carrier migration inside the material. Additionally, the breakdown strength of the composite was enhanced. It was proposed that the free volume of the nano-composites had no significant effect on the breakdown strength [24]. In summary, alumina nanoparticles could improve the thermal conductivity and dielectric performance of composites to a certain extent, however, the properties afforded by the composite could change dramatically depending on the different microscopic morphologies of the nano-fillers. Moreover, only a few studieshave studied the influence of alumina with different morphologies on epoxy composites. In this work, the thermal conductivity and the electrical performance of epoxy composites with aluminiumoxidenano-wires (Al_2_O_3_-NWs) wereinvestigated. Firstly, Al_2_O_3_-NWs were prepared by heating in a water bath, and Al_2_O_3_-NWs/epoxy resin composites with different doping ratios were prepared by blending the above-mentioned Al_2_O_3_-NWs with epoxy resins. The relationship between the thermal conductivity of the composites and the mass fraction of the fillers was analyzed. Overall, the effect of Al_2_O_3_-NWs on the electrical performance of composites, namely volume resistivity, dielectric performance, and breakdown performance, with different mass ratios was extensively investigated.

## 2. Materials and Methods

### 2.1. Materials

Bisphenol epoxy resin (Eponex 1513) E-51, anhydride curing agent, and methyltetrahydrophthalic anhydride were purchased from Changzhou Chongkai Chemical Co., LTD., China. 2,4,6-tri(dimethylamino) phenol (DMP-30) was purchased from Shanghai Aladdin Bio-Chem Technology Co., LTD., China. Aluminium sulfate octadecahydrate was purchased from Tianjin Damao Chemical Reagent Factory, China. Ammonium hydroxide was purchased from Chengdu Kelong Chemicals Co., Ltd., China.

### 2.2. Preparation of Al_2_O_3_-NWs and Epoxy Composites

A hydrothermal synthesis method was used for the preparation of Al_2_O_3_-NWs and epoxy composites. First, aluminum sulfate octadate was dissolved in deionized water, and the pH value of the solution was adjusted to 5 by adding ammonia to generate the precipitation of aluminum hydroxide. The mixed solution was placed in a high-pressure reaction kettle and heated in a water bath at 180 °C for 10 h. The formed solution was centrifuged to separate the aluminum hydroxide, which was cleaned with deionized water and alcohol, and then dried to obtain aluminum hydroxide powder. Finally, the powder was calcined at 600 °C for 2 h to obtain alumina nanowire powder.

Epoxy resin composites with different mass fractions of Al_2_O_3_-NWs were prepared by a blending technique. Firstly, the epoxy resin was heated at 60 °C in an oil bath until the viscous mixture became liquid. Then, different mass fractions of Al_2_O_3_-NWs (1, 3, 5, 7, and 10 wt%) were added to the epoxy resin matrix, and the mixture was stirred until evenly mixed. Methyl tetrahydrophthalic anhydride was added as the curing agent, which was dispersed and emulsified for 30 min. Lastly, 2,4,6-tri(dimethylamino) was added as an accelerant and stirred electrically for 30 min. The above mixture was placed in a vacuum box for degassing, then poured into a mold and heated for curing as follows: 80 °C for 1 h, 120 °C for 2 h, and 150 °C for 2 h [25].

### 2.3. Performance Analysis

#### 2.3.1. SEM Imaging

SEM was used to observe the microscopic morphology of Al_2_O_3_-NWs, as well as the dispersion of theAl_2_O_3_-NWs in the epoxy resin matrix. The samples were cooled with liquid nitrogen to make them brittle. The nanowires and the cross-section of the samples were sprayed with conductive gold, and the microscopic morphology was observed using an extremely narrow electron beam emitted from an SEM gun.

**Figure 1:**
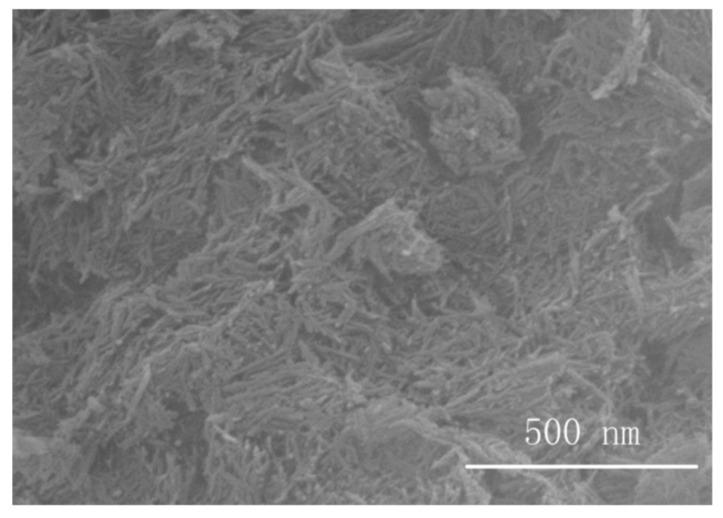
SEM images of nanowires.

Figure 1 shows the SEM image of the Al_2_O_3_-NWs. As observed, the alumina nano-fillers prepared via the hydrothermal method were linear, with a length of 100 nm and a diameter of 5 nm. The nanowires were well distributed with a clear interface between particles, the size was homogeneous, and no agglomeration was observed.

**Figure 2:**
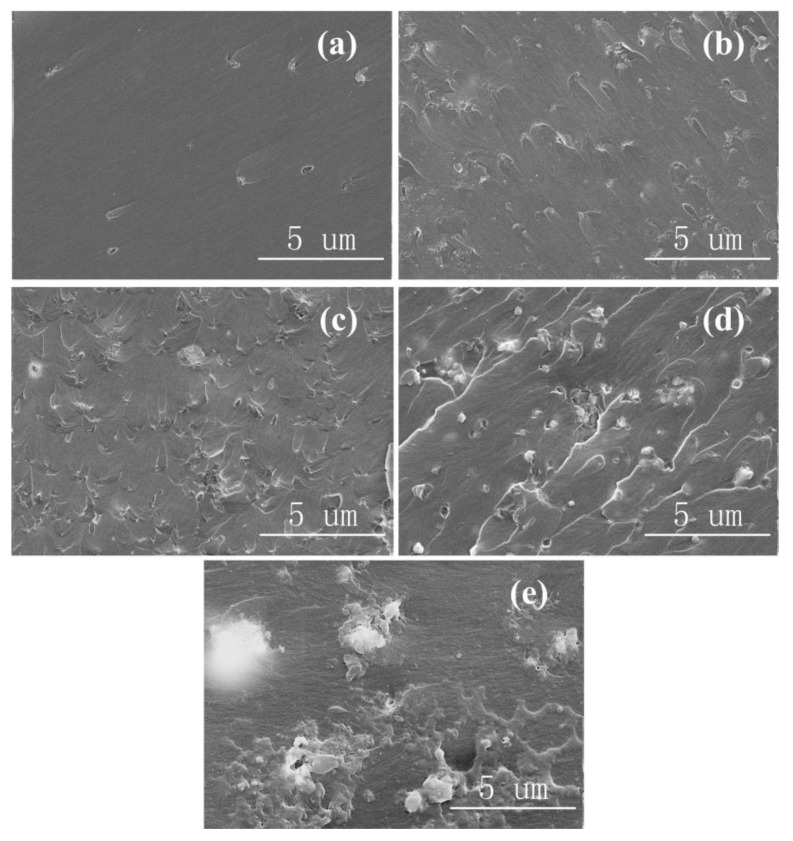
SEM images of Al_2_O_3_-NWs/epoxy resin composites with different mass ratios: (**a**) 1 wt%; (**b**) 3 wt%; (**c**) 5 wt%; (**d**) 7 wt%; and (**e**) 10 wt%.

Figure 2 shows the SEM images of the Al_2_O_3_-NWs/epoxy resin composites with different mass ratios. It can be observed that at low mass ratios, dot-shaped alumina fillers were coated and uniformly distributed throughout the epoxy resin matrix. The cross-section was shell-shaped with obvious characteristics of ductile fractures and good matrix/filler interfacial properties. As the mass fraction of fillers increased, the distance between alumina filler particles decreased and the matrix surface became rough with a large number of bumps, revealing that agglomeration occurred in the epoxy resin matrix and the alumina fillers overlapped each other to form a filler network.

#### 2.3.2. Thermal Conductivity

A multifunctional thermal conductivity meter (Xiangyi Instrument Limited Company, DRE-111, Xiangtan, China) was used to measure the thermal conductivity of the samples in a direction perpendicular to the composite plane by the Hot Disk Method. The transient plane heat source method was used for testing. The sample was heated by the Hot Disk probe, and the temperature change was calculated by the change of resistance inside the probe, so as to complete the measurement and transmission of temperature data.All the measured samples had a smooth and flat surface with a thickness of approximately 1 mm. The samples were stacked tightly and neatly and clamped to prevent the influence of air on the measurement of thermal conductivity. Graphite thin layers were sprayed on the surface of the measurement.We read and recorded the thermal conductivity obtained by the measuring instrument [26].

#### 2.3.3. Thermal Stability

Thermogravimetric Analysis-Differential Scanning Calorimetry(TG-DSC) was used to study the thermal stability of the composite materials. The weight of the sample was measured in a controlled heating environment as a function of temperature. In the experiment, 5 mg of the sample was added into a crucible, which was then placed in the apparatus. And nitrogen gas was introduced at a flow rate of 50 mL/min. The temperature was increased from 30 °C to 900 °C with a heating rate of 7 °C/min.

#### 2.3.4. Volume Resistivity

The volume resistivity of the composite was measured using a high insulation resistance tester (ZC36, Shanghai Anbiao Electronics Co., Ltd., China). The shield electrode, the protected electrode, and the measuring electrode were connected via a three-electrode connection method. A voltage of 1000 V was applied to both ends of the composite sample and the volume resistivity was recorded. In order to avoid interference of the polarized current and environmental factors on the experimental data, the volume resistivity was calculated by taking the average value of the resistivity with the voltage applied for 20 min. Finally, the volume resistivity of the composite sample was calculated using Equation (1):

(1) $$\rho \nu =R\nu \frac{Ae}{t}$$

where *ρν* is the volume resistivity of the sample, *Rν* is the volumeresistance of the sample, *Ae* is the effective area of the protected electrode, and *t* is the thickness of the sample.

#### 2.3.5. Dielectric Performance

Data such as the dielectric loss of the composite and its capacitance were measured by a high-frequency LCRdigital bridge (TH2826 by Tonghui Electronics Co., Changzhou, China). Before the measurement, the shield electrode, the protected electrode, and the measuring electrode were connected by a three-electrode connection method, where the shield and the protected electrodes were connected with the composite samples. Finally, data such as the gap and diameter of the measured electrode were recorded to calculate the dielectric constant. The dielectric constant was obtained from Equation (2):

(2) $$\varepsilon_{r}=\frac{4\times C\times h}{\pi \;(d_{1}+g{)}^{2}\varepsilon_{0}}$$

where *C* is the capacitance of the sample, *h* is the thickness, *d*_1_ is the diameter of the protected electrode, *g* is the electrode gap, and *ε*_0_ is the dielectric constant of vacuum. The frequency range of permittivity was 10–100,000 Hz, the test voltage was 0.5 V, and the number of scanning points was 800. The experiment was carried out at room temperature.

#### 2.3.6. Breakdown Strength

The breakdown strength of the composite was measured by breakdown voltage testing equipment controlled by a computer (Huabo Technology Industry Limited Company, Changchun, China, HJC-100KV). The composite sample was placed between spherical electrodes with a diameter of 25 mm. Transformer oil was used as the insulating dielectric. In the experiment, an AC voltage of 50 Hz was applied between the electrodes, and the voltage between the electrodesevenly increased at a rate of 1 V/s. Finally, the breakdown strength of the composite sample was calculated as the ratio of the breakdown voltage to the sample thickness.

## 3. Results and Discussion

### 3.1. Thermal Properties

**Figure 3:**
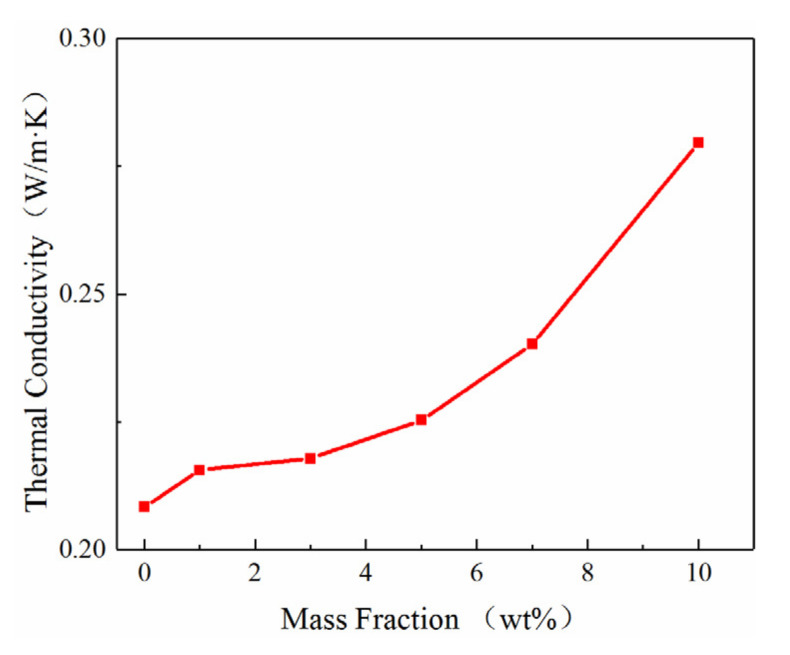
Thermal conductivity of Al_2_O_3_-NWs/epoxy resin composites with different mass ratios.

The results of the thermal conductivity tests of the pure epoxy resin and the Al_2_O_3_-NWs/epoxy resin composites with different mass ratios are shown in Figure 3. As can be observed, the thermal conductivity of the composites increased with the increasing mass fraction of Al_2_O_3_-NWs. Additionally, the thermal conductivity of the composites was better than that of the pure epoxy resin. When the mass fraction of Al_2_O_3_-NWs reached 10%, the maximum thermal conductivity was obtained, increasing by 34% compared with the pure epoxy resin, due to the better thermal conductivity of alumina compared with epoxy resin.

Low mass fractions of Al_2_O_3_-NWs fillers in the composite samples resulted in a low Al_2_O_3_-NWs density, leading to a low probability of contact between the particles. Therefore, the composite formed a dispersed heat-conducting chain structure inside. Compared with the pure epoxy resin, the thermal conductivity of the composite increased, but to a small extent. As the mass fraction of Al_2_O_3_-NWs increased, the density of the Al_2_O_3_-NWs also increased, leading to a high probability of contact between the wires. The increased density resulted in the nano-wires forming a series of thermally-conductive chains, thus creating a complex parallel thermal conductive network. At this time, the thermal conductivity of the composite material increased significantly, and it was significantly improved compared with that of the packing material with a lower mass fraction. When the mass fraction of the filler reached 10%, the thermal conductivity of the composite was 134% of that of the pure epoxy resin [27,28,29].

**Figure 4:**
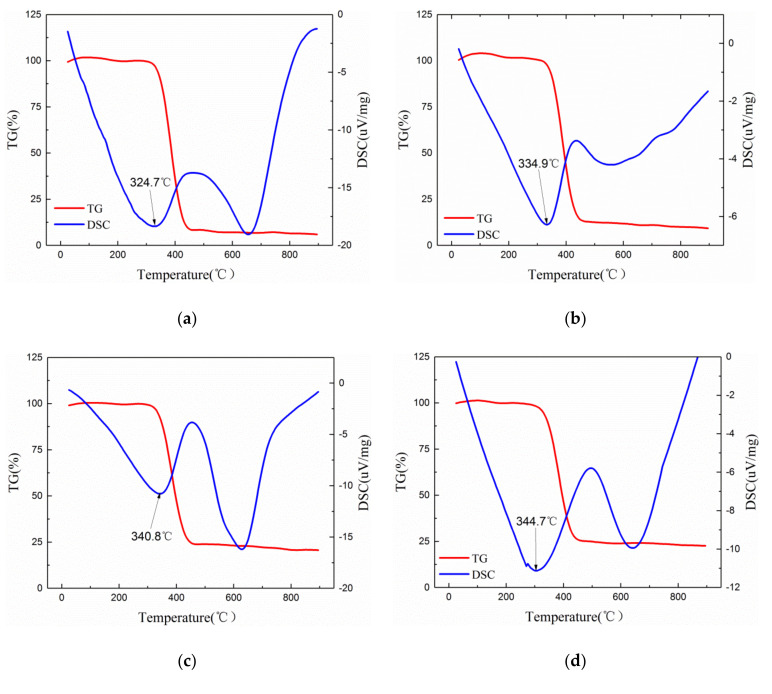

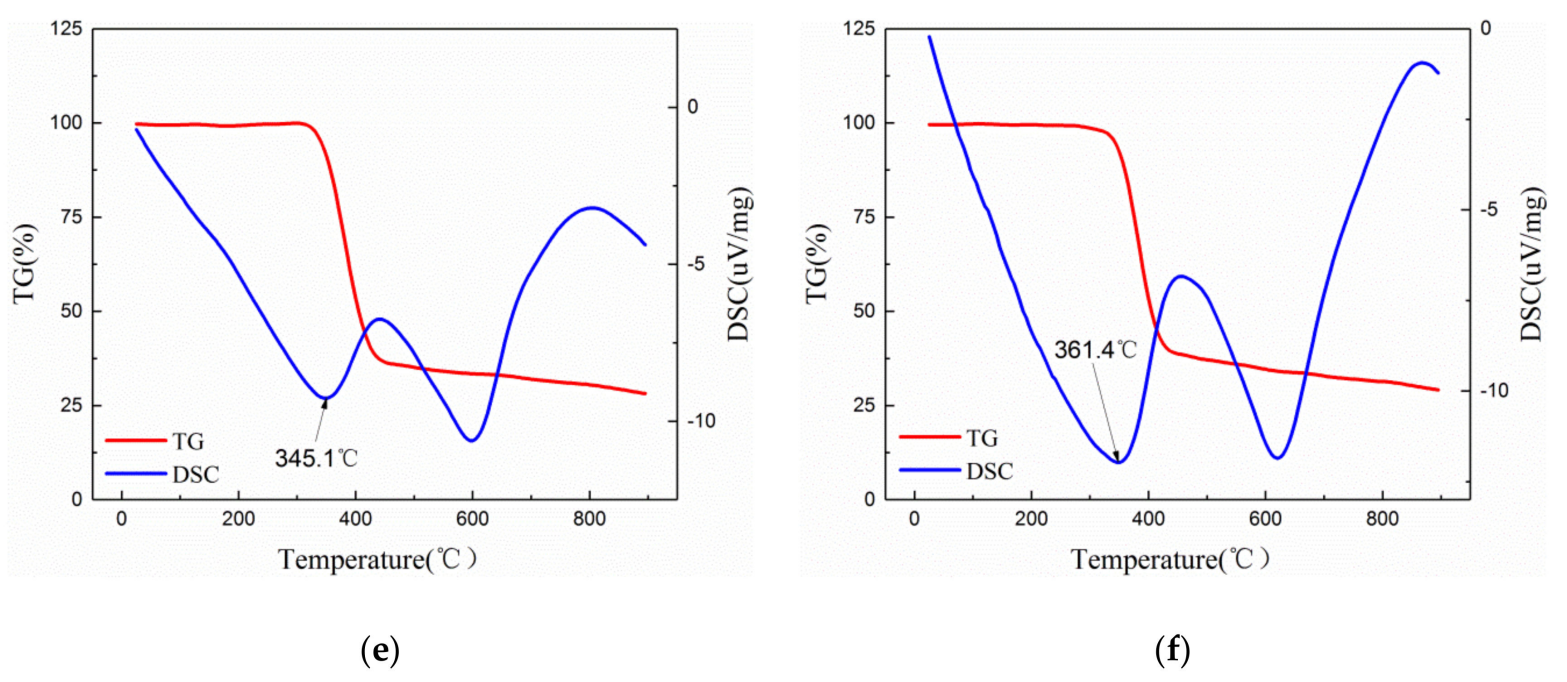
Thermogravimetric Analysis-Differential Scanning Calorimetry (TG-DSC) curve of Al_2_O_3_-NWs/epoxy resin composites with different mass ratios.(**a**) EP; (**b**) 1 wt%; (**c**) 3 wt%; (**d**) 5 wt%; (**e**) 7 wt%; and (**f**) 10 wt%.

Figure 4 is the TG-DSC curve of pure epoxy resin and theAl_2_O_3_-NWS/epoxy resin composite with different mass fractions.It can be seen from Figure 4 that the curve change trend of the six samples exhibited the sametrend within the test temperature range, indicating that the addition of Al_2_O_3_-NWS does not change the decomposition mechanism of the epoxy resin at high temperatures. The DSC curves of the samples have two obvious heat absorption peaks, but the TG curve only shows a significant change near the first heat absorption peak, indicating that at the temperature corresponding to the first heat absorption peak, the samples underwent thermal decomposition and the mass loss of the sample was accelerated and stabilized as the temperature increased. As can be seen from Table 1, with the increase of the mass fraction of Al_2_O_3_-NWS, the corresponding temperature (represented by T_10_ and T_50_ respectively) when the mass loss of the sample reached 10% and 50% also increases, reaching the temperature corresponding to the maximum thermal decomposition rate of the composite material. In addition, with the increase of the mass fraction of Al_2_O_3_-NWS, the temperature corresponding to the first heat absorption peak of the sample (represented by T_m_) increased, aswell as the thermal decomposition temperature. In summary, Al_2_O_3_-NWS was uniformly dispersed in epoxy resin and formed a thermal conductivity chain or network with the increase of mass fractions, which improved the thermal conductivity and enhanced the thermal stability of the composite material [30].

### 3.2. Volume Resistivity

**Figure 5:**
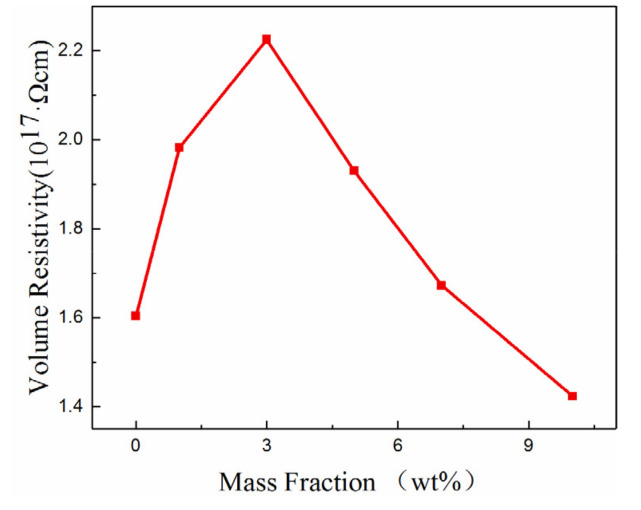
Volume resistivity of Al_2_O_3_-NWs/epoxy resin composites with different mass ratios.

The volume resistivity of the epoxy resin and the Al_2_O_3_-NWs/epoxy resin composites with different mass ratios were measured, and the results are shown in Figure 5.

It can be observed from the figure that the volume resistivity of the composites first increased and then decreased with increasing mass fraction of Al_2_O_3_-NWs, reaching a maximum value at approximately 3%. This may be due to the fact that when a small amount of Al_2_O_3_-NWs was added to the epoxy resin matrix, the wires bonded with the epoxy resin segments and the filler and matrix formed an interaction zone. The free volume of the molecules inside the composite decreased, which decreased the mobility of the carriers, and the volume resistivity of the composite increased. Additionally, when the mass fraction of the Al_2_O_3_-NWs was low, the addition of fillers introduced deep traps in the composite. These deep traps captured charges inside the composite and decreased the average displacement of the charged carriers, thereby inhibiting its movement. When the mass fraction of Al_2_O_3_-NWs increased, the space between nanowires decreased. Due to the high specific surface area of the incorporated Al_2_O_3_-NWs, agglomeration of Al_2_O_3_-NWs appeared in the composite, which reduced the effective interaction range and weakened the restraining effect on the carriers. At the same time, interaction regions overlapped with each other, providing a low-resistance path for charge carriers, which was beneficial for the transmission of carriers within the composite. Therefore, the volume resistivity of the composite increased [31].

### 3.3. Dielectric Performance

**Figure 6:**
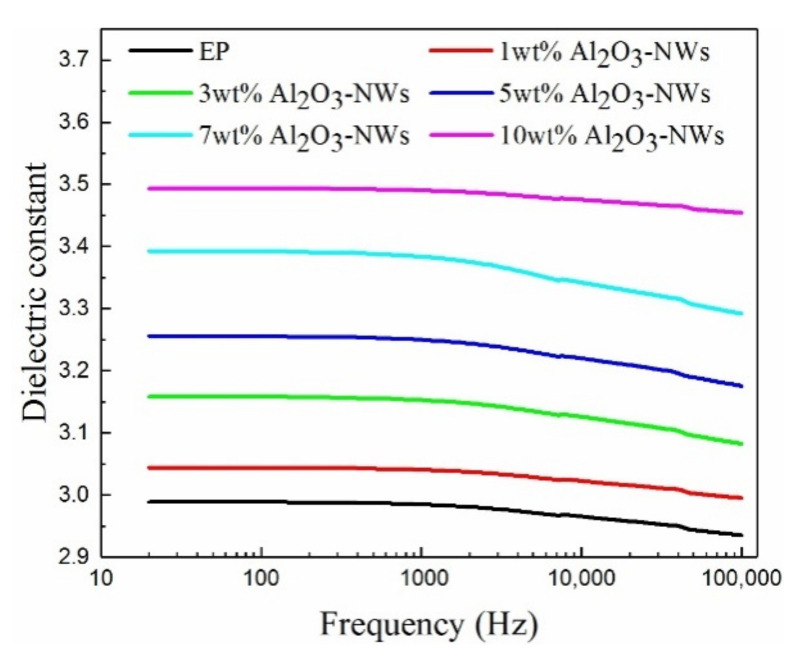
Dielectric constant of Al_2_O_3_-NWs/epoxy resin composites with different mass ratios as a function of frequency.

**Figure 7:**
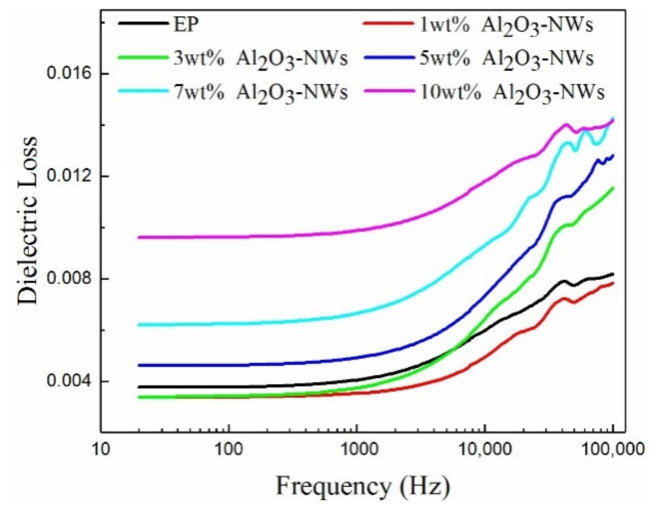
Dielectric loss of Al_2_O_3_-NWs/epoxy resin composites with different mass ratios as a function of frequency.

The dielectric constant and dielectric loss of the epoxy resin and the Al_2_O_3_-NWs/epoxy resin composites with different mass ratios were measured as a function of frequency, and the results are shown in Figure 6 and Figure 7.

The results reveal that the dielectric constant of the composites increased with increasing mass fraction of Al_2_O_3_-NWs, which was greater than the dielectric constant of the pure epoxy resin. This was likely because the dielectric constant of the Al_2_O_3_-NWs filler was approximately 10 [32], which was higher than that of the epoxy resin matrix, resulting in a higher dielectric constant of the composite compared with that of the pure epoxy resin. According to the Maxwell–Garnett formula, the dielectric constant of the composite can be expressed as [33]:

(3) $$k_{c}=k_{p}\left(1+\frac{\eta \rho_{p}f_{f}\left(k_{f}-k_{p}\right)}{\left(\rho_{p}f_{f}+\rho_{f}f_{p}\right)\eta k_{p}+\rho_{f}f_{p}\left(k_{f}-k_{p}\right)}\right)$$

where *f_p_* is the mass fraction, *k_p_* is the dielectric constant, and *ρ_p_* is the density of the epoxy resin; *f_f_* is the mass fraction, *k_f_* is the dielectric constant, and *ρ_f_* is the density of the Al_2_O_3_-NWs fillers; and *η* is a constant related to the shape and orientation of the fillers in the composite.

According to Equation (3), the dielectric constant of the composites is related to the matrix to filler doping ratio. The dielectric constant of the composite was calculated according to the formula, and it was observed that it increased with increasing mass fraction of Al_2_O_3_-NWs fillers, which was similar to the measureddielectric constant of the composite. The density of the polar groups in the composite increased with increasing mass fraction of nano-alumina fillers. This resulted in a higher number of introduced interfaces between the filler and the matrix, and the effect of interface polarization generated by the external electric field was enhanced. Therefore, the dielectric constant of the composite increased. With the enhancement of interface polarization, the charge distribution in the interface became denser due to the action of the interaction zone and the external electric field, which further increased the dielectric constant of the composite.

As observed, the dielectric constant of the composite decreased with increasing frequency, especially in the high-frequency region. This may be due to the heterogeneity of the composite. It was proposed that under an external electric field, free carriers in the composite were trapped by dielectric or interfacial traps during macroscopic movement, leading to a space charge accumulation in the interface region. Therefore, an electric dipole moment was formed in the region with uneven charge distribution. At low frequencies, dipole polarization was completed within the frequency variation cycle, with a stronger composite polarization performance and a larger dielectric constant. As frequency increased, the cycle shortened, and it was difficult to establish dipole orientation polarization. Therefore, the polarization performance of the composite decreased, as well as the dielectric constant.

As it can be observed from Figure 7, the dielectric loss of the composites decreased and then increased as the mass fraction of the Al_2_O_3_-NWs fillers increased. This may be due to the fact that at low Al_2_O_3_-NWs mass fractions, the matrix and nano-fillers were bonded tightly to form an interaction zone. Due to the special morphology of the nanowires, this interaction zone was interspersed in the epoxy resin matrix to form a network which restricted the movement of the polar groups and the epoxy resin segments within the composite, inhibiting the orientation polarization in the external electric field and reducing the dielectric loss of the composite. Moreover, when the mass fraction of Al_2_O_3_-NWs fillers increased, the filler density increased. Since the specific surface area of the nanowires was significantly larger than that of the nanoparticles, agglomeration in the composite increased, and the restriction effect on the polymer chain segment decreased. Additionally, the defects introduced by the agglomeration increased the conductive loss of the composite, so the dielectric loss of the composite increased accordingly [34].

Figure 7 reveals that the dielectric loss of the composite increased with increasing frequency, which may be due to the fact that this parameter is mainly affected byrelaxationpolarization and the conductive loss of the polar groups. When the temperature measured in the experiment was near room temperature, the conductive loss of the composite had a small effect on the dielectric loss, which was mainly due to relaxation polarization loss. At low external electric field frequencies, the polar groups in the composite were polarized under the external electric field, resulting in relaxation polarization loss. However, the dielectric polarization was able to follow the rapid oscillating external electric field, so the resulting relaxation polarization loss was low. As the frequency increased, the cycle of the external electric field was closer to, or shorter than, the cycle established by the internal relaxation polarization of the composite. Therefore, each delay in polarization would produce relaxation polarization loss, and increase dielectric loss of the composite. Additionally, due to the faster oscillating rate of the frequency in the high-frequency region, the rate of dielectric loss became larger.

### 3.4. Breakdown Strength

In this study, the Weibulldistribution was applied to analyze the data, which was embodied in the breakdown probability at a specific fieldstrength of the composite sample. For AC breakdown strength, the two-parameter Weibull distribution can be expressed as:

(4) $$P_{f}=1-e^{-{\left(\frac{E}{E_{0}}\right)}^{\beta }}$$

where *P_f_* is the accumulated breakdown probability of the sample; *E* is the breakdown strength; *E_0_* is the scaleparameter, indicating the breakdown strength when the probability of failure is 0.632; and *β* is the shapeparameter (positive), which reflects the breakdown voltage distribution range, meaning that the larger the *β*, the smaller the dispersion of breakdown voltage. In breakdown tests, according to the IEC/TC56 international standard, when the sample size is less than 25, *P_f_* can be expressed as:

(5) $$P_{f}=\frac{i-0.5}{n+0.25}$$

where *i* is the *i*th measured breakdown strength in ascending order, and n is the number of the measured breakdown strength [35]. From Equation (5), the number of permutations of the breakdown strength at a breakdown probability of 0.632 was obtained, and the corresponding breakdown strength is *E*_0_ in the Weibull distribution. Substituting *E*_0_ into Equation (4) with the measured breakdown strength, *E*, the *β* in the Weibull distribution was obtained.

**Figure 8:**
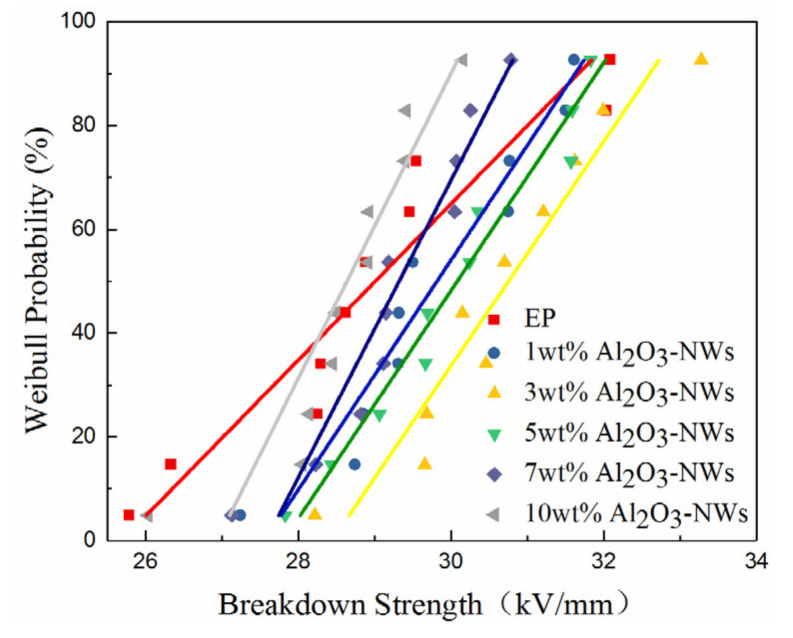
Weibulldistribution of the breakdown strengths of Al_2_O_3_-NWs/epoxy resin composites with different mass ratios.

Figure 8 reveals that the breakdown strength of the composites increased and then decreased with increasing mass fraction of the fillers, reaching its maximum at approximately 3%. From Table 2, we can see that the distribution coefficient of the composite was greater than that of the pure epoxy resin, and the composite presented a low AC impact dispersion.

It was hypothesized that when the mass fraction of Al_2_O_3_-NWs was low, Al_2_O_3_-NWs bondedtightly with the epoxy resin segments after being evenly dispersed in the resin matrix to form an interaction zone due to their large specific surface area. Therefore, the filler/matrix interface effect was enhanced, and deep traps were introduced to inhibit the movement of carriers in the composite. Additionally, the movement of polymer chains and space charges was restricted, which increased the breakdown strength of the composite. On the other hand, the composite generated significant heat accumulation under the external electric field during breakdown, which destroyed the insulation structure of the composite, resulting in breakdown of the composite. When the breakdown of the composite is under an AC electric field, the power loss per unit volume, *P*, can be expressed as:

(6) $$P=E^{2}\omega \varepsilon_{0}\varepsilon_{r}tan\delta_{0}e^{\alpha \left(t_{m}-t_{0}\right)}$$

where *ε_r_* refers to the dielectric constant of the material, tan *δ*_0_ is the tangent dielectric loss angle at 0 °C, α refers to the temperature coefficient of the tangent dielectric loss angle, *t_m_* is the maximum temperature of the dielectric, and *t*_0_ is the environmental temperature.

Additionally, most of the energy loss in dielectrics is converted to heat. For example, the dielectric loss will be converted into *Q*, which can be calculated as follows:

(7) $$Q=E^{2}h\omega \varepsilon_{0}\varepsilon_{r}tan\delta_{0}e^{\alpha \left(t_{m}-t_{0}\right)}$$

According to Equation (7), as the composite dielectric constant and dielectric loss of the compositeincreased, the heat generated inside the composite would also increase. However, at lowmass fractions of Al_2_O_3_-NWs, the dielectric loss of the composite at high frequencies decreased and the heat generated inside the composite was reduced. The thermal conductivity of the composite was largeand the heat accumulation in the dielectric decreased, which improved the breakdown strength of the composite.

When the mass fraction of the fillers increased, Al_2_O_3_-NWs were prone to agglomeration due to their large specific surface area, and the interaction zones overlapped with each other. This reduced the restriction on carriers, which was favorable for the formation of a low-resistance conductive pathway that resulted in the reduced breakdown strength of the composite. On the other hand, as the mass fraction of fillers increased, the dielectric constant and dielectric loss of the composite also increased. Moreover, the heat generated in the composite increased and it was more likely to accumulate inside the dielectric, which reduced the breakdown strength of the composite.

## 4. Conclusions

In this study, Al_2_O_3_-NWs/epoxy resin composites with different mass ratios were prepared. The effect of the addition of Al_2_O_3_-NWs on the thermal conductivity of the composites was investigated. The conclusions were as follows:

The thermal conductivity of the composites was enhanced compared with the bare epoxy resin. The thermal conductivity increased with increasing mass fraction of fillers. When the mass fraction of the fillers was 10%, the thermal conductivity of the composite was 34% higher than that of the epoxy resin. This was probably due to the fact thatAl_2_O_3_-NWs formed heat-conducting chains and networks inside the composite.

Additionally, it was determined that the presence of Al_2_O_3_-NWs can improve the insulating performance of the composite. The volume resistivity of the composite increased and then decreased as the mass fraction of Al_2_O_3_-NWs increased. When the mass fraction of the packing reached 3%, the internal agglomeration of the composite material was insignificant and the introduction of deep traps restricted the motion of carriers. Consequently, the volume resistivity reached a maximum value 38% higher than that of the epoxy resin.

The presence of Al_2_O_3_-NWs enhances the dielectric performance of composites. It was observed that the dielectric constant of the composite increased with increasing mass fraction of Al_2_O_3_-NWs, while its dielectric loss decreased and then increased as the mass fraction of Al_2_O_3_-NWs increased. As the frequency increased, the dielectric constant of the composites decreased, while the dielectric loss increased. This might be due to the interactions between the Al_2_O_3_-NWs and the epoxy resin matrix as well as the interface effect, both of which affects the polarization performance of the composite.

The presence of Al_2_O_3_-NWs can also improve the breakdown performance of the composite. The breakdown strength increased and then decreased as the mass fraction of Al_2_O_3_-NWs increased. When the mass fraction of the filler reached 3%, the volume resistivity of the composite material reached the maximum, and the dielectric loss was low. The heat accumulation inside the composite material was lower than that of samples with different mass fractions, and the AC breakdown strength reached the maximum.

## Figures and Tables

**Table 1:** TG-DSC test data of the pure epoxy resin and Al_2_O_3_-NWs/epoxy resin composites with different mass ratios.

Mass Fraction	EP	1 wt%	3 wt%	5 wt%	7 wt%	10 wt%
T_10_ (°C)	348.2	353.6	345.1	354.1	355.3	358.7
T_50_ (°C)	387.4	392.3	395.5	396.9	401.5	403.1
T_m_ (°C)	324.7	334.9	340.8	344.7	345.1	361.4

**Table 2:** Weibull distributions of scale and shape parameters of the pure epoxy resin and Al_2_O_3_-NWs/epoxy resin composites with different mass ratios.

Mass Fraction	*E*_0_	*β*
EP	29.45712	22.4857
1 wt%	30.74949	24.7283
3 wt%	31.20998	29.6959
5 wt%	30.35178	34.4664
7 wt%	30.04266	29.3359
10 wt%	28.91622	28.5079
